# Supplementary material for: Public awareness of acetaminophen and risks of drug induced liver injury: Results of a large outpatient clinic survey
Source: PLoS One. 2020 Mar 4;15(3):e0229070. doi: 10.1371/journal.pone.0229070 (PMC7055817; doi:10.1371/journal.pone.0229070)
Supplement: S1 Appendix — (DOCX) [file pone.0229070.s001.docx]

**Appendix 1 – Study Survey**

1. My gender is (please choose one):
   - Male
   - Female
2. My ethnic background is:

- Caucasian
- East/South East Asian (ie. China, Japan, Korea, Vietnam, Phillipines, Mayasia etc)
- South Asian (ie. Indo-Canadian)
- Middle-Eastern
- Hispanic (ie. Mexico, Central/South America etc)
- Indigenous
- African
- Other (please specify _______)

1. The city and country I was born in/at were __________, ___________
2. My highest level of education that I attended (may include partial attendance) ~~:~~

- Grade School
- High School or equivalent
- College/University
- Graduate School (or equivalent)
- Others (please specify _______)

1. Over the past year, I have used regular strength acetaminophen (ie. Tylenol products including generic) at this frequency:

- None
- Less than one day a week
- One to two days a week
- Three to five days a week
- Six to seven days a week

1. On the day I took regular strength acetaminophen/Tylenol, my average consumption over 24 hours was:

- 1-3 tablets/day
- Between 4-6 tablets/day
- Between 7-12 tablets/day
- More than 12 tablets/day

1. How often did I use extra-strength (ES) acetaminophen/Tylenol or Tylenol Arthritis in the last 12 months?

- None
- Less than one day a week
- One to two days a week
- Three to five days a week
- Six to seven days a week

1. On the days I used extra-strength acetaminophen/Tylenol, my daily consumption was:

- Less than 2 tablets a day
- 3-6 tablets a day
- 7-8 tablets a day
- 9-12 tablets a day
- More than 12 tablets a day

1. I take Advil or other non-steroidal anti-inflammatory drugs (i.e. Motrin, Ketoralac, Ibuprofen etc) at least once a week:

- True
- False

1. I usually drink alcohol

- I don’t drink at all
- I binge drink (i.e. many drinks all at once)
- Less than one drink/week
- 2-3 drinks/week
- 4-7 drinks/week
- 8-14 drinks/week
- More than 14 drinks/week

1. I think taking too much acetaminophen/Tylenol can be harmful to the following organ(s) (check all that apply):

- Not harmful
- Heart
- Liver
- Pancreas
- Stomach
- Brain
- Intestine/colon

1. I think taking acetaminophen/ Tylenol in combination with moderate amounts of alcohol is:

- Less harmful than excessive Tylenol use alone
- Equally harmful as excessive Tylenol use alone
- More harmful than excessive Tylenol use alone

1. I’m not sure Acetaminophen/Tylenol extra strength/arthritis products are:

- The same basic drug (ie. medical ingredient) as regular strength
- The same drug as regular strength but with an added drug that makes it work better
- A different drug than regular strength but made by the same company
