# Supplementary material for: Public awareness of acetaminophen and risks of drug induced liver injury: Results of a large outpatient clinic survey
Source: PLoS One. 2020 Mar 4;15(3):e0229070. doi: 10.1371/journal.pone.0229070 (PMC7055817; doi:10.1371/journal.pone.0229070)
Supplement: S2 Appendix — (DOCX) [file pone.0229070.s002.docx]

**Appendix 2 – Patient handout on Acetaminophen and Liver Toxicity**

Acetaminophen, also known as Tylenol^®^, is a widely available medication that is effective in relieving pain or fever. Acetaminophen is found in many common over the counter and prescription medications including Tylenol Arthritis^®^, Tramacet^®^, Oxycocet^®^, and many cough and cold preparations. Acetaminophen is generally considered safe when used according to the instructions on the medication’s labels. However, acetaminophen can cause serious liver damage if you take too much. Follow the following instructions to avoid acetaminophen related liver injury.

- Always read carefully the labeling information of medications that contain acetaminophen and follow dosing directions closely.
- Different over the counter products contain different amounts of acetaminophen. Always follow the dosing instruction on the label.
- Never take more than directed; even a small amount more than directed can cause liver damage.
- Don’t take more than one medicine that contains acetaminophen at a time.
- If you drink alcohol regularly (more than two drinks per day) or have a history of liver disease, speak to your doctor before taking acetaminophen as the toxic effects of acetaminophen, at levels that would ordinarily be safe, can become increased with alcohol consumption.
